# Buckling-Resistant and Trace-Stacked (BRATS) Design Enables Aid-Free Implantation of Flexible Multielectrode Array with Minimized Inflammatory Tissue Response

**DOI:** 10.1002/adfm.202512565

**Published:** 2025-07-28

**Authors:** May Yoon Pwint, Delin Shi, X. Tracy Cui

**Affiliations:** Department of Bioengineering, University of Pittsburgh, Pittsburgh, PA 15213, USA; Center for the Neural Basis of Cognition, Pittsburgh, PA 15213, USA; Department of Bioengineering, University of Pittsburgh, Pittsburgh, PA 15213, USA; Center for the Neural Basis of Cognition, Pittsburgh, PA 15213, USA; Department of Bioengineering, University of Pittsburgh, Pittsburgh, PA 15213, USA; Center for the Neural Basis of Cognition, Pittsburgh, PA 15213, USA; McGowan Institute for Regenerative Medicine, Pittsburgh, PA 15219, USA

**Keywords:** buckling-resistant design, in vivo microelectrode arrays, neural interfaces, thin-film microfabrication

## Abstract

Intracortical microelectrode arrays (MEAs) are vital tools for brain-machine interface applications and basic neuroscience research, with the potential to advance treatments for neurological disorders and enhance the understanding of the nervous system. However, implanting intracortical electrodes can damage native tissue along the insertion path and trigger inflammatory responses characterized by neuronal loss and glial activation. While flexible electrodes reduce some of these adverse effects compared to rigid counterparts, their mechanical compliance often leads to buckling during insertion, necessitating the use of insertion aids such as stiff shuttles or dissolvable coatings. These aids, however, introduce additional complexity and can cause further tissue damage. In this work, microfabricated polyimide MEAs featuring a buckling-resistant, trace-stacked (BRATS) design are presented. It is demonstrated that BRATS MEAs can penetrate agarose brain phantoms and the rat cerebral cortex without the need for insertion aids. Once implanted, BRATS MEAs established a stable and functional electrical interface with the brain, enabling high-fidelity, single-unit electrophysiological recordings. Compared to conventional flexible MEAs inserted with a stiff shuttle, BRATS MEAs elicited significantly lower inflammatory responses and preserved a higher density of neurons near the implant site one week post-implantation.

## 1. Introduction

Advances in neural engineering and neuroscience enable technologies for treatment of various neurological disorders such as Parkinson’s disease, epilepsy, and tetraplegia.^[1–3]^ One such enabling technology is intracortical microelectrode arrays (MEAs). Intracortical MEAs enable high-resolution interfacing with the brain.^[4]^ However, implantation of such an MEA inevitably disrupts the native brain tissue, destroys the cells along the path of the implant, ruptures the cell membranes and vasculature, and deforms the cells nearby.^[5–7]^ Disruption of the dense vascular network in the brain and rupture of the blood-brain barrier (BBB) result in deposition of plasma proteins such as fibrinogen and accumulation of iron, leading to neurotoxicity and increases in reactive oxygen species (ROS).^[7–9]^ ROS then causes secondary injury by oxidizing lipids and proteins and downregulating tight junction proteins, leading to BBB damage and the release of pro-inflammatory cytokines.^[7,10]^ The implantation of an MEA also directly and indirectly affects the neuronal health near the implant, rupturing and deforming the neurons.^[5,11]^ The insertion damage also activates microglia, followed by astrocytes, resulting in a glial sheath around the implant.^[5,12,13]^

In addition, tissue strain from device implantation contributes to implantation injury and chronic tissue reaction around the implant.^[5,7,14]^ Intracortical MEAs fabricated on stiff substrates such as silicon cause higher mechanical strain on the tissue due to the mechanical mismatch between the implant and the brain tissue, and continuously irritate the surrounding tissue through micromotions.^[5,7]^ On the other hand, flexible MEAs fabricated on polymers such as polyimide are mechanically compliant, allowing them to move with the micromotions of the brain, leading to better tissue integration.^[5]^ Therefore, for chronic implantation, flexible electrodes cause less strain on the surrounding tissue, and soft material-based electrodes are preferred for reduced immune reaction.^[15]^ However, soft and flexible electrodes are difficult to implant because they are mechanically compliant and often buckle when inserted into the brain before penetrating through the pia mater. Many workaround strategies have been developed such as insertion shuttles, guides, stiffening coatings, liquid metal stiffeners, shape memory polymers, magnetic guidance, and microfluidic actuation.^[16–24]^ Unfortunately, the setups to apply those methods can be very cumbersome, and many of the approaches cause extra insertion trauma to the brain. In addition, targeting may be difficult since the inserted probes could move in the case of shuttle retraction or dissolution of stiffening material.^[20]^ These issues become more problematic for multi-shank MEA devices. Hence, there is a crucial need for flexible MEAs capable of penetrating the brain without external aid.

In this work, we designed polyimide intracortical microelectrode arrays that can be implanted without additional assistive devices. We engineered the cross-section of the MEA by stacking two layers of electrode traces in the center, which increased the channel count without increasing the probe width while maintaining the minimal cross-sectional area. The buckling-resistant and trace-stacked (BRATS) design allows for aid-free insertion, thereby minimizing the insertion trauma and reducing the implant footprint. Furthermore, due to the simplicity of the surgical procedure, the implantation of multiple shank MEAs is easily scalable. We demonstrated that the BRATS MEAs do not buckle during insertion, and they can establish a functional interface with rat cortical tissue acutely after implantation. We compared BRATS to the conventional flat design and found that BRATS design elicits significantly less inflammatory response and results in a higher neuronal population near the implant 1-week post-implantation. This histological finding is supported by the ability of the BRATS MEA to record high-fidelity single-unit activities from the rat cortex.

## 2. Results and Discussion

### 2.1. Buckling Load and Cross-Sectional Area Calculations

**Figure 1.**
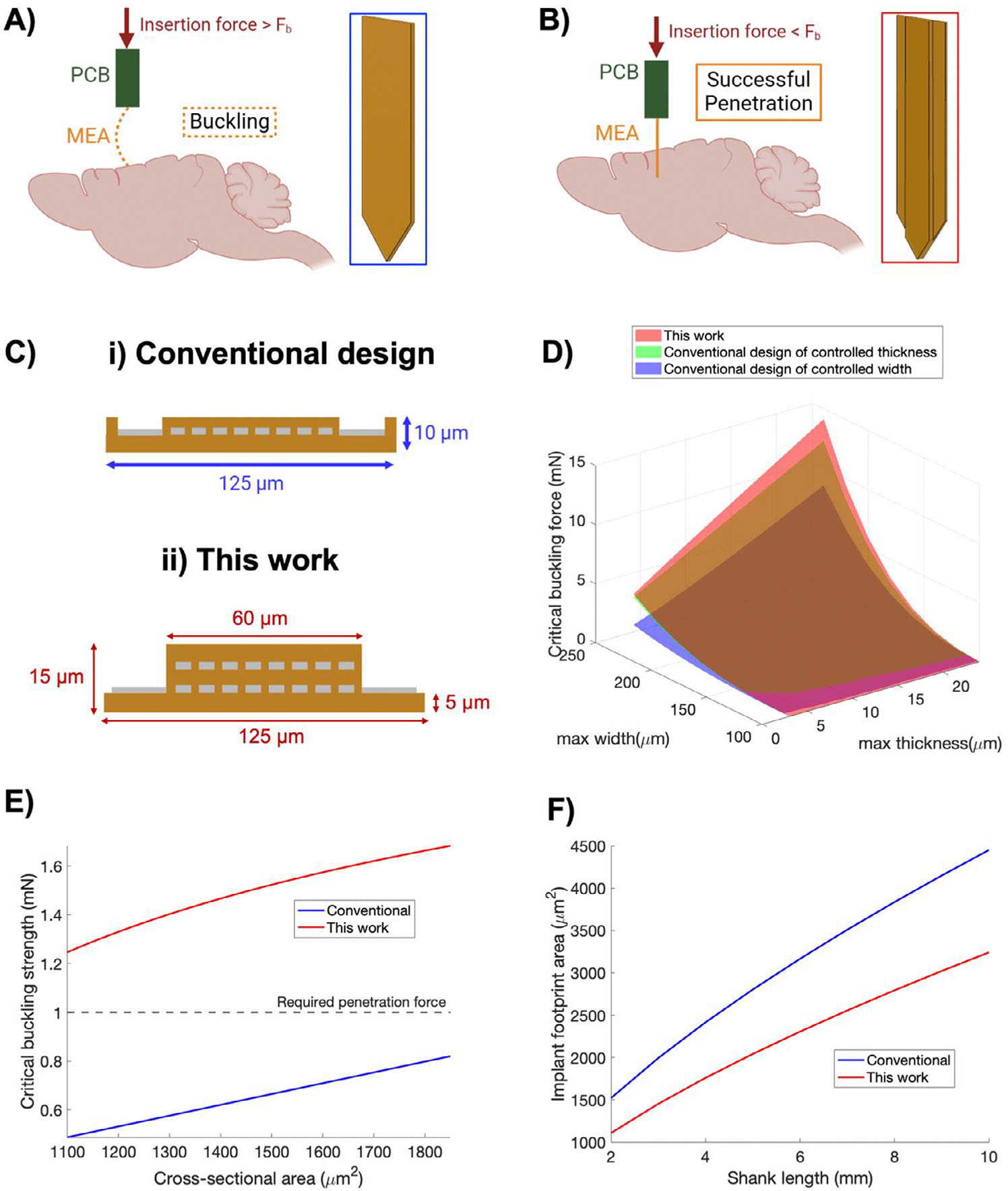
Illustration of the BRATS MEA design and working principle. A) MEA buckling when insertion force is larger than critical buckling force. Inset: Perspective view of a conventional flexible MEA design. B) Successful penetration of MEA into the brain when insertion force is less than critical buckling force. Inset: Perspective view of a BRATS MEA designed in this work which can penetrate the brain. C) Cross-sectional views of conventional (top) versus BRATS (bottom) designs and the dimensions used for comparisons. D) Effects of changing geometric parameters on the critical buckling strength on BRATS (red) and conventional designs (green and blue) while keeping the area constant. E) Difference in critical buckling strength between conventional and BRATS designs with different cross-sectional implant footprint areas. F) Minimum cross-sectional area needed to achieve buckling-resistant insertion for conventional versus engineered design at different shank lengths. Schematics created with BioRender.com.

Using Young’s modulus *E* = 5.39 GPa estimated from nanoindentation experiments (Figure S4, Supporting Information) and *K* = 0.5, *L* = 2 mm, and *F*_*B*_ = 1 mN, the minimum required moment of inertia can be computed by rearranging Equation 1. However, since the moment of inertia depends on multiple geometric parameters, such as width and thickness in the case of conventional rectangular cross-section MEAs, the minimum required moment of inertia alone is not enough to determine the parameters in this under-constrained problem. As such, we will constrain the maximum width of the probe to be between 100 and 250 μm. For conventional design, this then results in required total thicknesses approximately between 9.7 and 13 μm, corresponding to cross-sectional areas of 1312 and 2416 μm^2^, respectively. For the BRATS probes, we have two additional geometric parameters. Suppose the maximum width of the BRATS MEA to be between 100 and 250 μm, the stem width *b* is 60 μm (Figure 1C), and the probe has three uniform insulation layers, we have a minimum required thickness of each layer between 4.65 to 4.20 μm.

Taking these into consideration, we designed our BRATS MEAs to have a flange thickness h of 5 μm, flange width *B* of 125 μm, stem width b of 60 μm, and total thickness *H* of 15 μm (Figure 1C; Figure S2B, Supporting Information) which has a cross-sectional area of 1225 μm^2^ and buckling strength of 1.35 mN.

To compare the buckling performance of the new design to the conventional rectangular cross-section design, we will keep the implant footprint area (i.e., cross-sectional area) constant and further constrain the design parameter space. Suppose that the stem width *b* is 60 μm, and three uniform layers each 5 μm in thickness. By keeping the area constant and varying the width between 100–250 μm and thickness between 3–16 μm, we can determine the critical buckling force as shown in Figure 1D. Here, note that the conventional design (green and blue curves) never exceeded the buckling strength of BRATS MEAs designed in this work (red).

Suppose again that the maximum width is between 100 and 250 μm, the stem width *b* is 60 μm, and three uniform layers each 5 μm in thickness and cross-sectional area for each comparison is constant, we can compute the corresponding thickness of the conventional MEA as well as the resulting moments of inertia for both designs. By extension, if we assume *E* = 5.39 GPa, *K* = 0.5, and *L* = 2 mm, we obtain the critical buckling strengths illustrated in Figure 1E. Using the above-mentioned parameters, the BRATS MEA design has an average of 2.3-fold higher critical buckling strength than the conventional design.

Assuming the same parameters, if we then study the cross-sectional areas required to achieve buckling-free insertion for both designs, the conventional design always requires a larger implant footprint (without assistive shuttles) at any length of the MEA between 2 mm and 10 mm (Figure 1F).

### 2.2. Micromotion-Induced Tissue Strain

**Figure 2.**
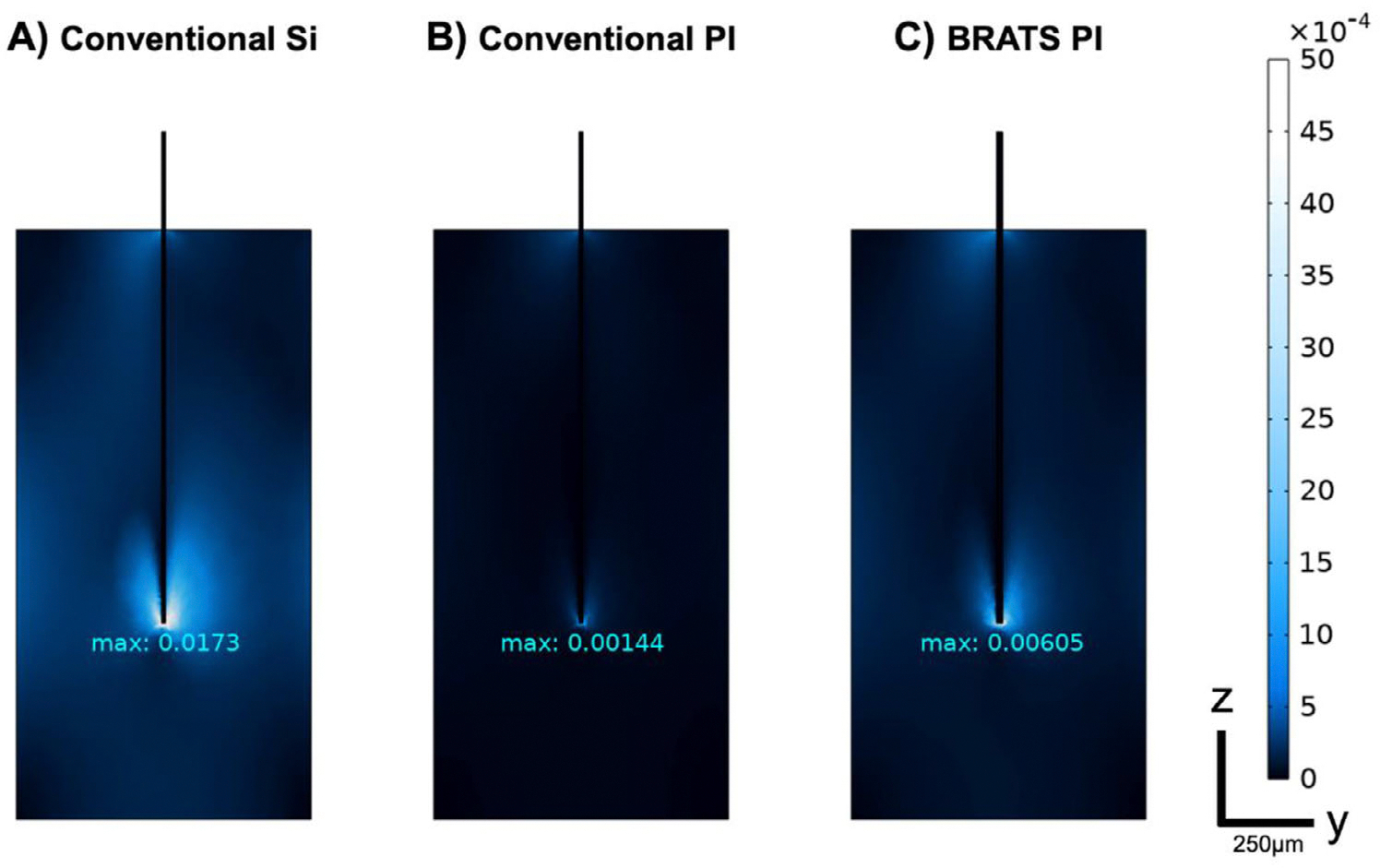
Finite element simulation of tissue strains due to micromotion. Cross-sections showing first principal strain around A) conventional silicon, B) conventional polyimide, and C) BRATS MEA when displaced 1 μm tangentially in y direction. Same first principal strain (dimensionless) color scale. Scale bar = 250 μm in y and z.

Previous simulation and in vivo studies have shown that micromotions arising from heartbeat and breathing induce strain around tethered intracortical MEAs and that the stiffer the MEA is, the higher the strain.^[5,37,42]^ Our finite element simulation results show strain profiles as expected, with the maximum strain being at the tip of the MEAs (Figure 2). During a tangential displacement along the axis of buckling Δy = 1 μm, we found the maximal volumetric strain at the tip of conventional silicon MEA to be 1.7%, while changing the material to flexible polyimide results in a 92% reduction (0.14%). The BRATS design resulted in 0.6% maximum volumetric strain at the tip, higher than the conventional polyimide design, but still a 67% reduction than the stiff silicon MEA (Figure 2).

### 2.3. Fabrication and Characterization

**Figure 3.**
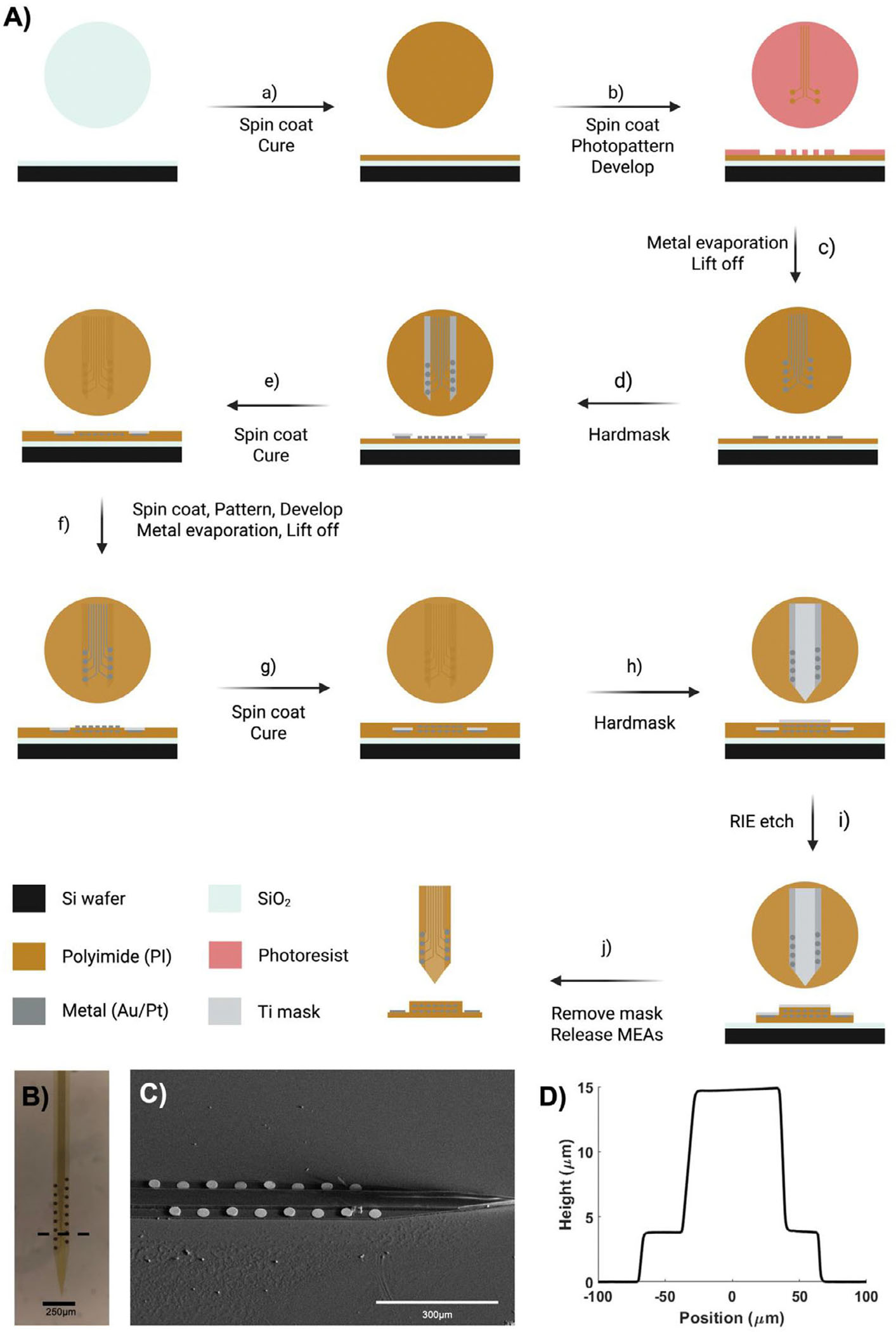
Microfabrication and characterization of the BRATS MEA. A) Schematics of the fabrication process. B) Optical image of the BRATS MEA. C) Scanning electron micrograph of a representative MEA seen from 45-degree angle. D) Surface profile of the BRATS MEA taken across the dotted line shown in B.

The MEAs were fabricated using dimensions in Figure 1C and representative optical and electron micrographs illustrate the design of the engineered cross-section (Figure 3; Figure S2B, Supporting Information). While the BRATS design and the second metal layer add some complexity to the fabrication process, we designed our etch masks such that one RIE etch step forms all the electrodes and the MEA shape. This eliminates the need for repeated masking and etching of individual polyimide layers as well as stripping of the masks. In addition, our choice of titanium as the etch mask, along with a silicon dioxide sacrificial layer, enabled combined stripping of the etch mask and release of the MEAs via a single final buffered oxide etch at the same time. Although the deposition of a second metal layer incurs additional cost, it doubles the channel count, and the BRATS design overall eliminates the cost of shuttle assembly and complex surgery typically required for conventional flexible MEAs. The fabricated BRATS MEAs are optically semi-transparent, and the stem is barely visible (Figure 3B). The 45° titled view under scanning electron microscope reveals the profile of the flange and stem as well as the electrodes that are flush to the surface (Figure 3C). Surface profilometry also shows the flushness of the electrodes as well as the geometric profile of the MEA (Figure 3D).

In addition, we characterized the electrochemical impedance spectroscopy (EIS) and obtained 1 kHz impedance of 323 ± 13kΩ (mean ± SEM [standard error of the mean]). From the cyclic voltammogram, we also computed charge storage capacity (CSC) from 16 sites of a representative MEA and obtained a mean cathodic CSC of 10.2 ± 1.1 mC/cm^2^ and a total mean CSC of 15.2 ± 0.7 mC/cm^2^ (mean ± SEM, Figure S4B, Supporting Information).

### 2.4. Insertion Tests

**Figure 4.**
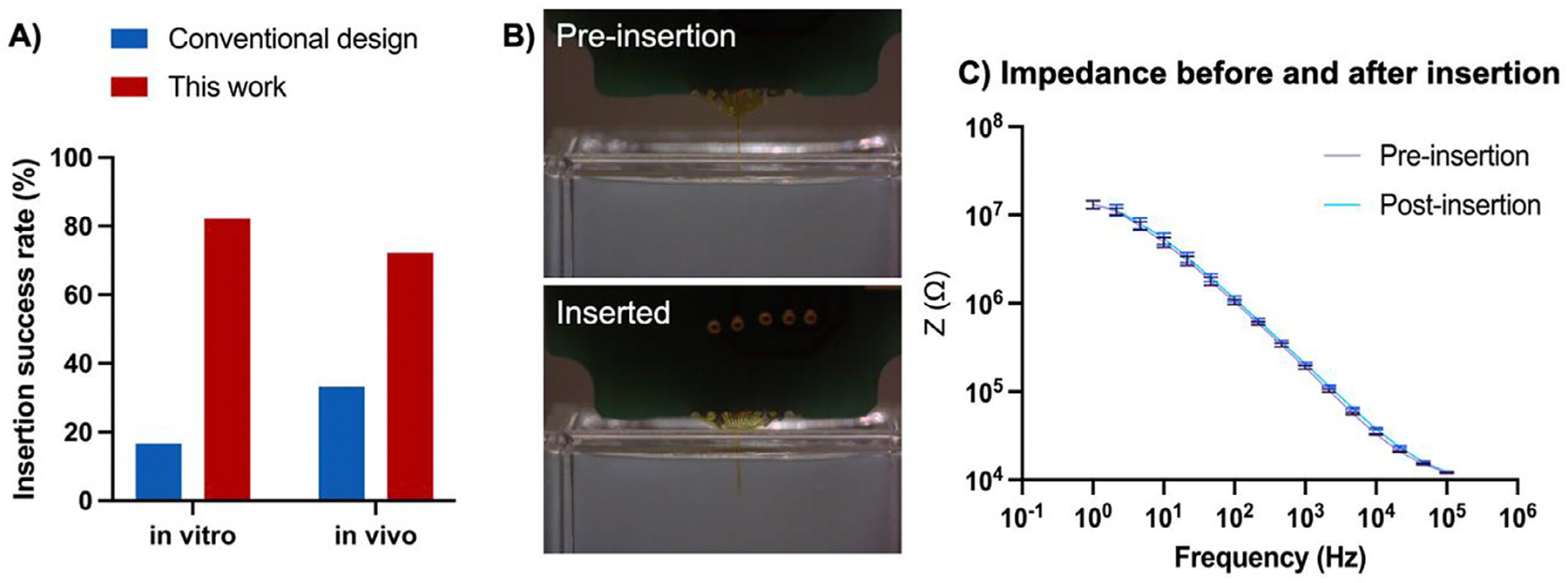
Demonstration of insertion in the agarose brain phantom and in rat brain. A) Insertion success rates of MEAs with conventional (n = 12 in vitro, n = 12 in vivo trials total) versus engineered (n = 28 in vitro, n = 18 in vivo trials total) designs inserted without any secondary assistance. B) Intra-operative optical images of in vitro insertion. C) EIS of a representative MEA before and after insertion (n = 16 sites, mean ± SEM).

The BRATS MEAs were subjected to insertion tests in vitro using 0.6% agarose gel brain phantom (Figure 4). Each MEA was inserted into the gel, and for control, the conventional planar MEAs with the same cross-sectional area were used (Figure 1C). The BRATS MEAs with engineered cross-section were able to insert over 80% of the trials while the conventional design can penetrate the gel in less than 20% of the trials (Figure 4A). Optical images of an MEA before and during insertion are shown in Figure 4B. The same insertion test performed in a rat brain also shows a higher success rate for the engineered design than the conventional design. In addition, we demonstrated that the insertion also did not result in any significant change in EIS in vitro (Figure 4C).

### 2.5. In Vivo Electrophysiology in Rat Brain

**Figure 5.**
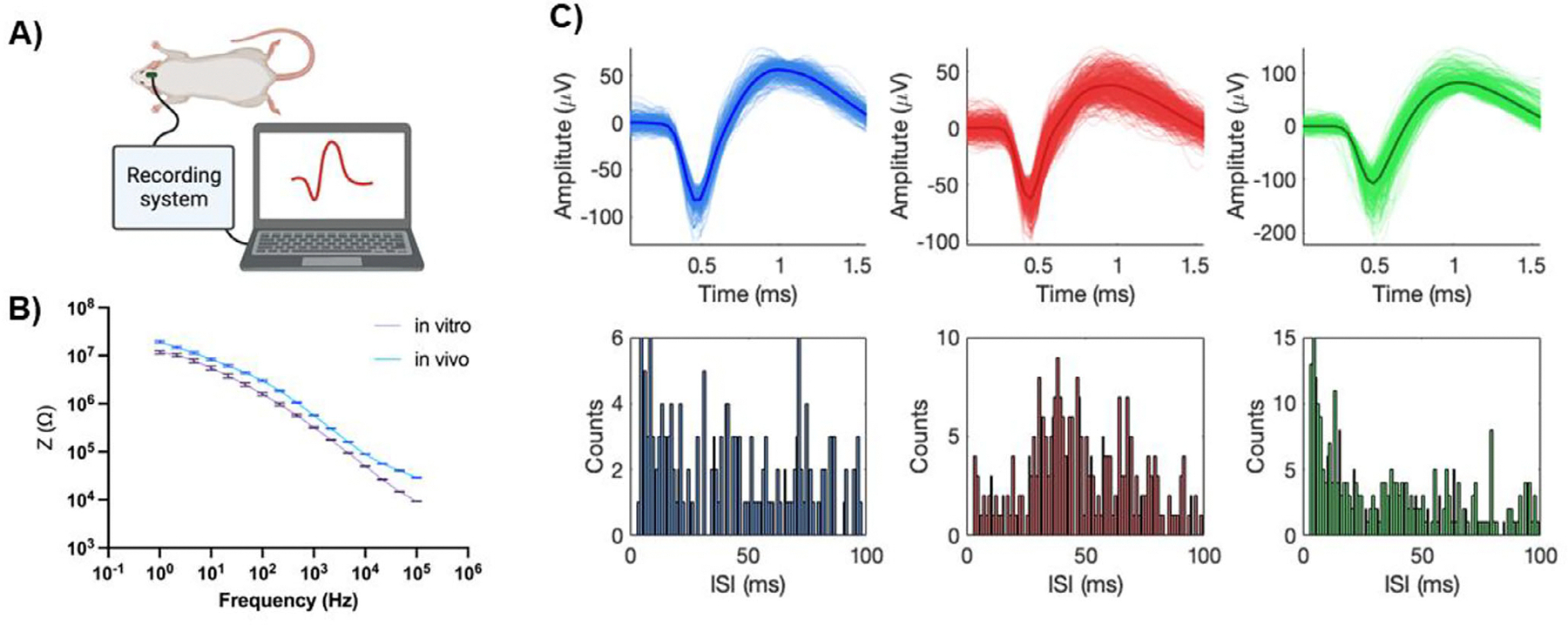
Electrophysiological functionality of the BRATS MEA. A) Illustration of the experimental setup where electrophysiologicalcal activities were recorded from the rat cortex. B) EIS of the MEA before and after implantation (n = 16 sites, mean ± SEM). C) Distinct single unit waveforms (top) and their interspike intervals (ISIs) (bottom) from one electrode site ≈625 μm deep in the cortex.

Conventional flexible MEAs that rely on an insertion shuttle, injection needle or stiffening coatings often do not get high yield recording immediately after implantation, due to the shuttle-induced injury. For studies using these probes, recordings usually start after a recovery time (1–2 weeks).^[29,43,44]^ Here we demonstrate high-yield recording on day 0 to indicate the benefit of the BRAT design. We implanted the BRATS MEA in the cortex of a Sprague Dawley rat and recorded acute electrophysiological activities (Figure 5A). Electrochemical impedance spectroscopy measurements taken before and after implantation show a mean impedance increase of 251 ± 9.4 kΩ at 1 kHz in vivo (mean ± SEM, Figure 5B), which is consistent with the literature.^[45]^ This is due to the differences between 2- and 3-electrode setups as well as the tissue resistance in vivo.^[46]^ Extracellular voltage changes from neuronal spiking activities were also recorded from the lightly anesthetized rat and the spikes were sorted using principal component analysis and adaptive K-means using Plexon Offline Sorter. Three distinct single units sorted from the deepest electrode site (≈625 μm deep) are shown in Figure 1C. The MEA recorded single and multi-unit activities from 15 sites (93.75% yield) with an average of 2.56 units per channel and signal-to-noise ratio (SNR) of 6.47±1.40 (mean±SEM) (Figure S6, Supporting Information). The high yield and high SNR acute recording implies establishment of a seamless tissue-device interface immediately following implantation, highlighting an advantage over conventional flexible MEAs, which undergo a recovery period of 1–2 weeks due to the insertion aid induced trauma.

### 2.6. Early Chronic Tissue Response

**Figure 6.**
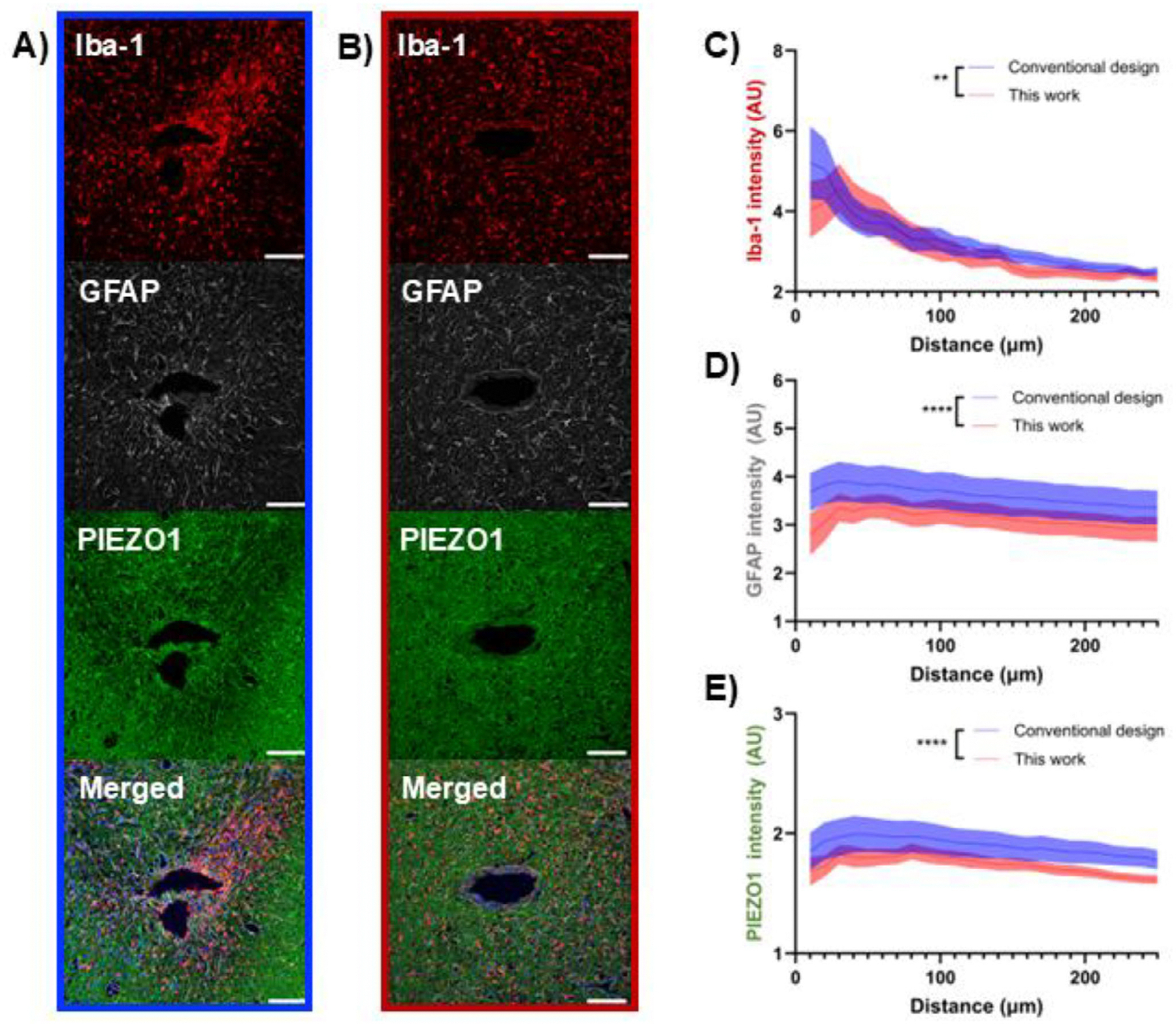
Glial and mechanosensitive response to conventional and BRATS MEAs. Immunofluorescent images of the tissue around A) conventional and B) BRATS MEA after 1 week stained for PIEZO1 (green), Iba-1 (red), and GFAP (gray). Merged images include DAPI in blue channel. All scale bars 100 μm. Quantification of C) PIEZO1, D) Iba-1, and E) GFAP intensities (arbitrary units, AU) normalized to the four corners of each image. Statistical analyses were conducted using Wilcoxon signed rank test where * p < 0.05, ** p < 0.01, and **** p < 0.0001. Data presented as mean ± SEM.

To determine the potential benefit of our design on inflammatory response after 1 week, we evaluated the glial scarring and mechanosensitive response of the BRATS MEAs compared to conventional flat design (Figure 6). We stained for Iba1 (ionized calcium-binding adaptor molecule 1) as a marker for microglia and macrophages, and GFAP (glial fibrillary acidic protein) as a marker for astrocytes; both markers are upregulated when activated during inflammation. We found that overall Iba-1 and GFAP expressions are higher for the conventional design when comparing distance-matched fluorescent intensities within 250 μm from the probe hole (Wilcoxon signed rank test, Figure 6C,D). The stronger inflammatory glial response at this time point is likely due to the additional stab injury caused by the tungsten shuttle required for the insertion of conventional MEAs.^[47]^

In addition, we used PIEZO1 to study the response of cells near the implants in response to mechanical stress. PIEZO1 is a mechanically sensitive ion channel protein whose expression is upregulated due to mechanical stress in the local environment. Our static mechanics simulations showed that the BRATS design exerts higher mechanical strains than conventional design, we wondered if this increased strain would elicit a stronger PIEZO1 response. Interestingly, we found lower PIEZO1 activity around the BRATS MEA, which could be due to the PIEZO1 upregulation in activated microglia and astrocytes around the conventional MEA due to the insertion shuttle (Figure S7, Supporting Information). Lower PIEZO1 activity around BRATS MEAs could suggest better wound healing and device integration.

Prior studies showed that polymer MEAs improve tissue integration compared to silicon MEAs, especially at longer timepoints.^[29,48,49]^ These studies suggest that although the polymer MEAs require an insertion shuttle, the stab injury caused by the shuttle may recover over time. A longer-term histological study is warranted to examine if BRATS and conventional MEA implants induce different mechanosensitive responses, after the stab wound response has been completely resolved.

**Figure 7.**
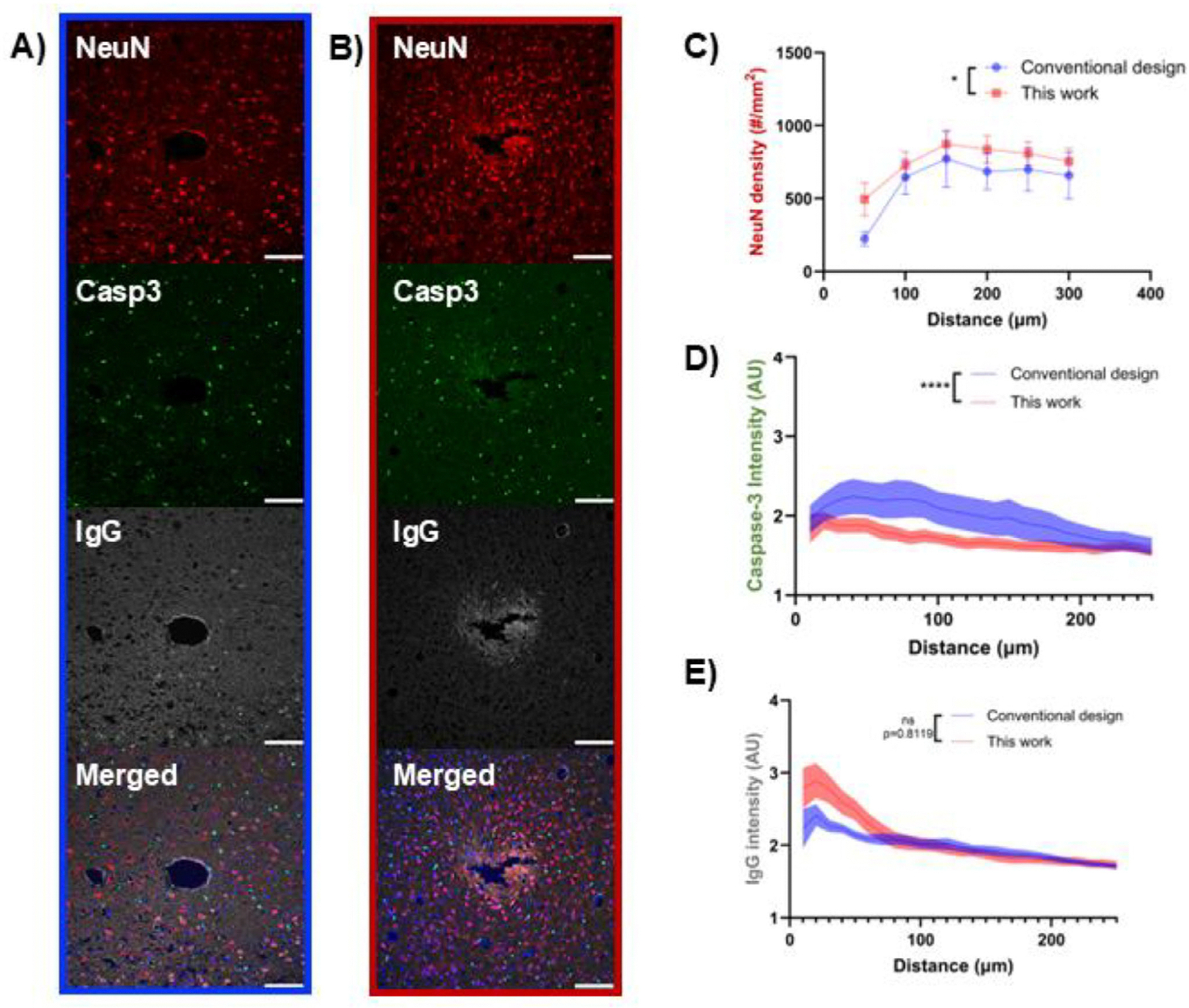
NeuN, Caspase-3, and IgG expression around conventional and BRATS MEAs. Immunofluorescent images of the tissue around A) conventional and B) BRATS MEA after 1 week stained for Caspase-3 (green), NeuN (red), and IgG (gray). Merged images include DAPI in blue channel. All scale bars 100 μm. C) Density of NeuN-positive cells, D) Intensity quantification Caspase-3, and E) Intensity quantification of IgG as a function of distance from MEA track. Statistical analyses were conducted using the Wilcoxon signed rank test where * p < 0.05 and **** p < 0.0001. Data presented as mean ± SEM.

To further elucidate the tissue response, we studied the apoptotic cell death, neuronal density, and BBB leakage around the MEAs (Figure 7). Neuronal soma stain NeuN showed higher neuronal density overall within 300 μm of the BRATS MEAs compared to the conventional flat design (Figure 7C). On the contrary, there was higher apoptosis around conventional MEAs using Caspase-3 as a marker for apoptotic cell death (Figure 7C). This could be due to the greater extent of microgliosis in shuttle-assisted flat MEAs, which could displace the neuronal population near the implant. However, we found no statistically significant difference in IgG, a marker for BBB leakage, between the two designs (Figure 7E).

Overall, we found lower Caspase-3 intensity and higher NeuN density as well as lower glial response in 1-week histology. These histological results indicate better tissue integration, which could translate to better recording performance, especially in the early chronic phase. We expect that a better outcome in the early chronic phase will result in better chronic performance. However, it is important to conduct chronic studies to assess long-term performance.

## 3. Conclusion

We presented the design and fabrication of buckling-resistant, trace-stacked (BRATS) polyimide microelectrode arrays (MEAs). These BRATS MEAs can be implanted into the rodent brain without the need for stiff shuttles or coatings, while enabling higher channel counts and minimizing the device footprint. The trace-stacking approach allows for the integration of additional electrode traces without increasing the device width, and the reduction in individual polymer layer thicknesses maintains overall dimensions optimized for buckling resistance. While the multi-layer fabrication approaches have been demonstrated previously, ^[29,39,50–54]^ BRATS MEA design features the smallest cross-section among all reported buckling-resistant, insertion-aidless polymer devices, to our knowledge.

We demonstrated the functionality and effectiveness of the BRATS MEAs through insertions into brain phantom gels and live rat brains, as well as single-unit recordings in the rat cortex. Compared to conventional flexible MEAs inserted with a shuttle, BRATS MEAs caused less neuronal damage and elicited lower acute inflammatory responses. The elimination of the shuttle not only avoids shuttle-induced injury but also simplifies the implantation procedure—an especially important advantage for MEAs with multiple shanks. Overall, the BRATS design represents a promising platform for high-resolution, large-scale mapping of electrophysiological activity across brain regions.

## 4. Experimental Section

### Microelectrode Array (MEA) Design and Working Principle:

The most common mechanism via which a flexible MEA shank fails to penetrate into the brain is structural buckling (Figure 1A). Buckling was a result of a slender structure unable to support the applied longitudinal force. The buckling of a flexible probe could be modeled by Euler buckling equation^[25]^:

(2) $$F_{B}=\frac{\pi^{2}EL}{{\left(KL\right)}^{2}}$$

where

$F_{B}$ = critical buckling force,

E = modulus of elasticity,

I = moment of inertia,

L = length of the probe, and

K = effective length factor.

During implantation of a penetrating microelectrode array into the brain, the MEA experiences ≈1 mN of force.^[26]^ Therefore, for a successful insertion, it needs $F_{B}\;\geq \;1\;mN$. As described in Equation 1, critical buckling force $F_{B}$ is a function of Young’s modulus or modulus of elasticity E, moment of inertia or second moment of area I, and effective length K.L.

Young’s modulus is a fundamental property of a material related to the stress-strain response of the material.^[27]^ To simplify the calculations and design considerations, we assume that the modulus of the MEA is primarily determined by the insulating material. This is because the insulation layer materials far exceed the materials in the conductive layers in quantity, with each insulating film measuring on the order of micrometers in thickness, whereas the conductive layers are typically only nanometers thick. Some of the commonly used biocompatible polymer-based insulators for neural interfaces include polyimide, parylene-C, and SU8.^[28–32]^ Although Young’s modulus is a fundamental property, varying results have been reported for these materials.^[31–35]^ The values can drastically vary depending on the fabrication methods and environmental factors such as specific compositions, temperature, and UV exposure.^[33–35]^ Therefore, to accurately reflect the material properties after being subject to specific microfabrication processes, Young’s modulus values of the thin polymer films were measured experimentally via nanoindentation. Specifically, Young’s modulus was computed from the load versus displacement curve of nanoindentation using Triboscan 9 software. Among the three biocompatible polymers mentioned, polyimide was chosen in this work due to its high thermal stability, high dielectric strength, chemical resistance, and low water absorption.^[25,36]^

During the implantation of penetrative microelectrodes, the connector end was fixed to a stereotaxic manipulator, and the tip of the MEA should touch the brain surface before penetration (Figure S1, Supporting Information). When the insertion force was applied to the MEA, there were three possible buckling scenarios giving rise to three different theoretical K values.[17,27] Since the connector end was affixed to a stereotaxic frame, no rotation or lateral translation could occur at the fixed end. According to the surgical setup described above, the most likely scenario was that the penetrating end will be translation and rotation fixed (Figure S1A, Supporting Information) since it will be touching the brain right before the start of insertion. In this case, the theoretical K value was 0.5. If, however, the electrode was allowed to move or rotate, the MEA shank will have a free end leading to a higher K value of 1 and lower buckling strength (Figure S1B, Supporting Information). In an improper setup where the electrode does not touch the brain surface, the K value will be 2 (Figure S1C, Supporting Information). Therefore, proper surgical setup was crucial for successful insertion and surgery will proceed only if everything was set up correctly. Thus, it will assume K=0.5 for design calculations.

Related to the coefficient K is the length of the MEA L. According to Equation 1, the critical load decreases with the square of the length. Therefore, shank length of 2 mm was chosen to constrain the design space. Since the goal here was to design an intracortical electrode for a rat model, a 2 mm long shank was sufficient.

The last parameter in the Euler buckling equation is the moment of inertia I, also known as the second moment of area. I depends on the size, shape, and geometry of the probe.[25] Conventionally, the moment of inertia is described by:

(2) $$I=\frac{wt^{3}}{12}$$

where w = width of the MEA and t = thickness of the MEA (Figure S2A, Supporting Information).

In this work, the cross-section of the MEA was designed such that the moment of inertia was larger for the same cross-sectional area (Figure 1C; Figure S2, Supporting Information). The moment of inertia, in this case, was a function of the width of the flange B, the width of the stem b, the total thickness H, the thickness of the flange h, and the location of the centroid $y_{c}$ as seen in (Figure S3, Supporting Information) and can be computed by the following equation (See Figure S3, Supporting Information for derivation):

(3) $$I=\frac{Bh^{3}}{12}+Bh{\left(y_{c}-\frac{h}{2}\right)}^{2}+\frac{b{\left(H\;-\;h\right)}^{3}}{12}+b(H-h){\left(y_{c}-\frac{H\;+\;h}{2}\right)}^{2}$$

### Finite Element Analysis of Tissue Strain around the MEAs:

To study how the BRATS MEA design would affect the surrounding tissue strain, a finite element analysis in COMSOL Multiphysics (COMSOL Inc., Stockholm, Sweden) and compared it to conventional design of the same material (polyimide, E = 5.39 GPa) and stiffer material (silicon, E = 200 GPa ^[37]^) was conducted. It was built 3D models of the MEAs (Figure 1A,B) in Fusion 360 (Autodesk, San Francisco, CA). The 3D models were then imported into COMSOL Multiphysics. A 0.75 × 0.75 × 1.5 mm^3^ block was constructed as a model for the brain, with Young’s modulus of 5.51 kPa.^[37]^ Using the Solid Mechanics module, the first principal strain when the bottom surface of the brain was displaced 1 μm laterally (Δx = 1 μm) and tangentially (Δy = 1 μm) while the top surface of the MEA was fixed, simulating brain micromotions with tethered intracortical microelectrode implant was studied.

### Microfabrication of the MEAs:

MEAs were fabricated on a 4-inch silicon wafer with 500 nm thick SiO_2_ layer (University Wafers Inc, Boston, MA). The wafer was first cleaned with acetone, isopropyl alcohol (IPA), and deionized (DI) water, sequentially, then dried and heated on a hot plate at 150 °C for 5 min and treated with O_2_ plasma using a reactive ion etcher (RIE, Trion Phantom III LT, Trion Technology) for 1 min at 200 mTorr and 150 W. The cleaned wafer was then spin-coated with polyimide (PI) precursor PI-2525 (HD Microsystems LLC, Parlin, NJ) at 2500 rpm for 1 min and soft baked at 120 °C and 150 °C for 30 s each (Figure 3A-a). The polyimide coated wafer was then cured in a programmable tube furnace (STF1200, Across International, Livingston, NJ) in a nitrogen (N_2_) environment at 350 °C for 1 h.

Next, platinum (Pt) electrodes were patterned by photolithography and a metal lift-off process. A bi-layer photoresist process was used for metal lift-off. First, the PI wafer was plasma treated in 50 sccm O_2_ at 200 mTorr and 150 W for 60 s to improve adhesion of the subsequent metal layer. Next, LOR-5B (Kayaku Advanced Materials, MA) was spun on the wafer at 4000 rpm for 90 s and soft-baked at 195 °C for 9 min. MICROPOSIT S1805 (Kayaku Advanced Materials, MA) was then spun at 5000 rpm for 90 s and soft-baked at 120 °C for 5 min. The wafer with bi-layer photoresist was then exposed to 365 nm ultraviolet (UV) light using a maskless aligner (MLA, MLA100 Heidelberg Instruments, Switzerland) with a dose of 50 mJ cm^2^. After UV exposure, the wafer was developed in 351 Developer 1:4 (Kayaku Advanced Materials, MA) and AZ400K Developer 1:4 (MicroChemicals GmbH, Germany) for 75 and 45 s, respectively (Figure 3A-b). The wafer was cleaned with deionized (DI) water and dried with N_2_ subsequently. Following that, a mild O_2_ plasma cleaning was done at 600 mT and 60 W for 60 s to descum and further clean the surface for metal deposition. A metal stack (20 nm Pt – 100 nm Au – 30 nm Pt) was evaporated on the wafer using an electron beam evaporator (Plassys MEB550S, Angstrom Engineering) and then the metal was lifted-off in remover PG (Kayaku Advanced Materials, MA) to define the electrodes, metal traces, and connection pads (Figure 3A-c).

The wafer is then cleaned with acetone, IPA, and DI water sequentially and dried with N_2_ flow. To define the flanges of the MEA, a layer of titanium (Ti) mask was patterned using the bi-layer photolithography process (Figure 3A-d).

Oxygen plasma treatment was done in RIE at 200 mTorr, 150 W for 90 s. A second layer of PI-2525 was spun on top following the procedures described above and cured in N_2_ at 340 °C for 1 h (Figure 3A-e). A second layer of Pt electrodes was also patterned using the same procedure as above (Figure 3A-f). The third and final layer of PI was deposited again via spin coating and cured in N_2_ at 330 °C for 1 h (Figure 3A-g). A final Ti mask was patterned in the shape of the stem using the bi-layer photolithography process (Figure 3A-h). The MEAs were then formed by reactive ion etching in oxygen plasma at 200 mTorr and 150 W (Figure 3A-i). Subsequently, a mild 2-min argon (Ar) plasma cleaning was done at 40 mTorr and 15 W with 40 sccm Ar, and an additional 20 nm of Pt was deposited to form pristine Pt electrode sites. Finally, the MEAs were released in buffered oxide etch (BOE 7:1, Transene Company, Inc., Danvers, MA) which removes both the Ti mask and the sacrificial SiO_2_ layer (Figure 3A-j).

### Animal Surgery:

All animal procedures were approved by the Institutional Animal Care and Use Committee of the University of Pittsburgh. Sprague-Dawley rats (both male and female, Charles River Laboratories, Garfield Heights, OH) were anesthetized with isoflurane (5% for induction, 2%–3% for maintenance), and placed on a thermal pad. After induction, the head was fixed into a stereotaxic frame (Kopf Instruments, Tujunga, CA). A midline incision was made in the scalp using a #11 scalpel blade and connective tissue and blood were removed using cotton swabs. The scalp was retracted, and burr holes were drilled in the skull between the bregma and lambda to expose the brain, for insertion tests.

For the electrophysiology experiments, a 2.3 mm diameter burr hole was drilled at 7.0 mm posterior and 5.0 mm lateral from bregma. Using a bent 29-gauge needle, the dura mater in the cranial window was carefully resected, avoiding visible damage to the brain tissue and major blood vessels.

For the immunohistology assessment of the device tissue interface, we implanted two MEAs bilaterally (one BRATS and one conventional design) in each male Sprague-Dawley rat (n = 4, 250–350 g, Charles River, Wilmington, MA). The MEAs were implanted ≈4.5 mm posterior and ±3 mm Iateral to bregma while avoiding visible blood vessels. The conventional MEAs were inserted using a 50 μm tungsten wire shuttle (Advent Research Materials Ltd, Eynsham, England) attached with polyethylene glycol (Sigma–Aldrich, St. Louis, MO) solution 30% w/v. Standard aseptic surgical procedures were used as approved by the Institutional Animal Care and Use Committee of the University of Pittsburgh. A midline incision was made in the scalp and connective tissue was removed, and burr holes were drilled in the skull between bregma and lambda. Additional burr holes were made for bone screws to anchor the head cap afterward. The MEAs were implanted and secured with Kwik-Sil (World Precision Instruments, Sarasota, FL) and blue-light curing dental cement. Once the head cap was fully secured, the scalp was sutured back together.

### Insertion Tests:

Insertion tests were performed using BRATS and conventional designs, as illustrated in Figure 1C. The probes were connected to a custom printed circuit board (PCB) via a zero-insertion force connector (Hirose Electric, Kanagawa, Japan). The PCB/MEA assembly was then mounted onto a stereotaxic manipulator and set to touch the phantom or brain surface before insertion. For in vitro tests, the brain phantoms were made by dissolving 0.6% agarose in hot DI water and letting it gel at room temperature before use. For in vivo tests, the surgery was performed on a female Sprague-Dawley rat as described above. Care was taken to avoid repeated insertion into the same site. In all insertion tests, the MEAs were inserted 1.5 mm deep from the surface at a speed of 100 μm s^−1^ using the Neural Glider Inserter without ultrasonic actuation (Actuated Medical, Bellefonte, PA).

### Electrochemical Characterization:

Electrochemical characterizations were performed using a potentiostat/galvanostat (Autolab, PGSTAT128N, Metrohm). Cyclic voltammetry (CV) was performed by sweeping the working electrode potential between 0.8 and −0.6 V at a scan rate of 100 mV s^−1^. For in vitro tests, Ag/AgCl electrode was used as the reference and Pt wire as the counter electrode in 1 x phosphate buffered saline (PBS). For in vivo characterization, a subcutaneous Ag/AgCl wire was used as the reference electrode and a stainless-steel bone screw was used as the counter electrode. Charge storage capacity was estimated by integrating the current versus potential CV curve and dividing it by scan rate and electrode area to obtain the charge per area.^[38]^ Electrochemical impedance spectroscopy (EIS) was done by superimposing a sine wave (10 mV RMS amplitude) onto the open circuit potential while varying the frequency from 1 to 10^5^ Hz to characterize the electrode/electrolyte interface.^[38,39]^

### Electrophysiological Recording:

A male Sprague-Dawley rat underwent surgery as described above and was implanted with a BRATS MEA described in this work. After surgery, isoflurane was reduced to ≈1.5% to maintain a lightly anesthetized state. Electrophysiological activity was recorded from the MEA at 24 414 Hz (RX7, Tucker-Davis Technologies Inc., Alachua, FL) and analyzed in the spike sorting software Offline Sorter (Plexon Inc., Dallas, TX).

### Immunohistochemistry:

Surgical procedures were performed as described above for bilateral implantation of non-functional MEAs for histological studies. After 1 week of implantation, the rats were sacrificed and transcardially perfused with PBS, followed by 4% paraformaldehyde. The brain was then extracted and dehydrated in 15% and 30% sucrose, sequentially. The removed brain tissue was cryoprotected using optimal cutting temperature compound (OCT, Fisher HealthCare, Houston, TX), frozen, and the cortex was sectioned horizontally down to 1050 μm. The tissue sections were subdivided into two groups. One group was stained for mechanosensitive ion channels (1:250 mouse anti-PIEZO1, Novus Biologicals, Centennial, CO), microglia (1:500 rabbit anti-Iba1, Wako Chemicals, Richmond, VA), and astrocytes (1:500 chicken anti-GFAP, MilliporeSigma, Darmstadt, Germany). The other group was stained for neuronal cell body (1:500 mouse anti-NeuN, MilliporeSigma, Darmstadt, Germany), apoptotic cell death (1:500 rabbit Asp175, Cell Signaling Technology, Boston, MA), and blood-brain barrier injury (1:500 goat anti-rabbit IgG, Invitrogen, Carlsbad, CA). Both groups were counter-stained with DAPI for cell nuclei. The tissue sections were imaged using a confocal laser scanning microscope Fluoview FV3000 equipped with a 20x air objective at the Center for Biologic Imaging at the University of Pittsburgh.

To quantify the PIEZO1, Iba-1, GFAP, Caspase-3, and IgG immunofluorescent staining, pixel-based image intensity analytics were performed using previously published custom MATLAB script I.N.T.E.N.S.I.T.Y. v2.0.^[14]^ For each channel in each image, the background was calculated as the average of 5% of each of the four corners. To calculate the background noise intensity threshold, pixels with an intensity less than Mean+1 × Standard Deviation were considered tissue holes and removed from the calculation. The dimensions of the MEA tracks were approximated as 125 μm × 15 μm. Using MATLAB, the center of the MEA track was identified on each image, after which the script generated masks of 25 concentric rings, each ring 10 μm wider than the previous. The average intensity for all pixels above the background noise intensity threshold in each bin was calculated and normalized against the 5% background to calculate the Signal-to-Noise Intensity Ratio (SNIR) in each bin. The SNIRs were plotted against distance from the MEA track in Figure 5 and Figure 6.

For the quantification of neuronal cells, we used the deep neural network-based algorithm Cellpose (www.cellpose.org) to segment the NeuN channel. We then performed human-in-the-loop training using 8 images (4 from conventional and 4 BRATS MEAs).^[40]^ The generated cell masks from both training and test images were then imported into Fiji.^[41]^ From Fiji, we obtained the coordinates of the center of mass (CoM) of each segmented cell. It was then used the I.N.T.E.N.S.I.T.Y script to generate 50 μm masks around the MEA track and computed the number of neurons in each bin using CoM locations. Neuronal density was then computed as the number of neurons in each bin divided by the area of the corresponding bin.

### Statistical Analysis:

Statistical comparison between conventional design and BRATs were performed using Wilcoxon matched pairs test on each stain. Then post hoc analyses were done using multiple Wilcoxon matched pairs tests with Holm-Sidak correction in GraphPad Prism.

## Supplementary Material

supplementary info

Supporting Information

Supporting Information is available from the Wiley Online Library or from the author.

## Data Availability

The data that support the findings of this study are available from the corresponding author upon reasonable request.
