# Supplementary material for: Buckling-Resistant and Trace-Stacked (BRATS) Design Enables Aid-Free Implantation of Flexible Multielectrode Array with Minimized Inflammatory Tissue Response
Source: Adv Funct Mater. Author manuscript; Available in PMC 2026 Jun 27. (PMC13309171; doi:10.1002/adfm.202512565)
Supplement: supplementary info [file NIHMS2177492-supplement-supplementary_info.docx]

**Supplementary Information**

**Buckling-resistant and Trace-stacked (BRATS) Design Enables Aid-free Implantation of Flexible Multielectrode Array with Minimized Inflammatory Tissue Response**

May Yoon Pwint^1,2^, Delin Shi^1,2^, X. Tracy Cui^1,2,3, #^

1. Department of Bioengineering, University of Pittsburgh. Pittsburgh, PA,15213, USA.
2. Center for the Neural Basis of Cognition, Pittsburgh, PA, 15213, USA.
3. McGowan Institute for Regenerative Medicine, Pittsburgh, PA 15219, USA

# Corresponding Author: xic11@pitt.edu


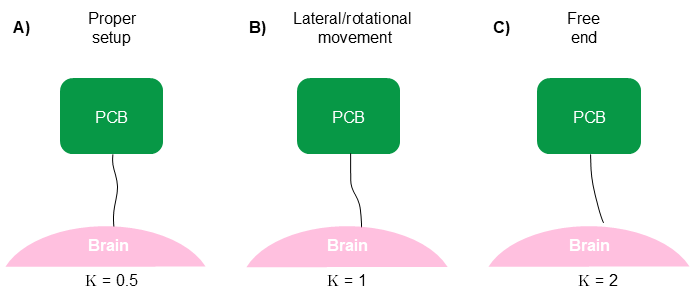
Figure S1: Illustration of how the effective length factor K changes depending on the boundary conditions. A) Proper setup when the MEA is touching the brain before insertion. B) MEA experiences lateral/rotational movement due to improper setup. C) MEA is not touching the brain surface leading to a boundary condition with free end.


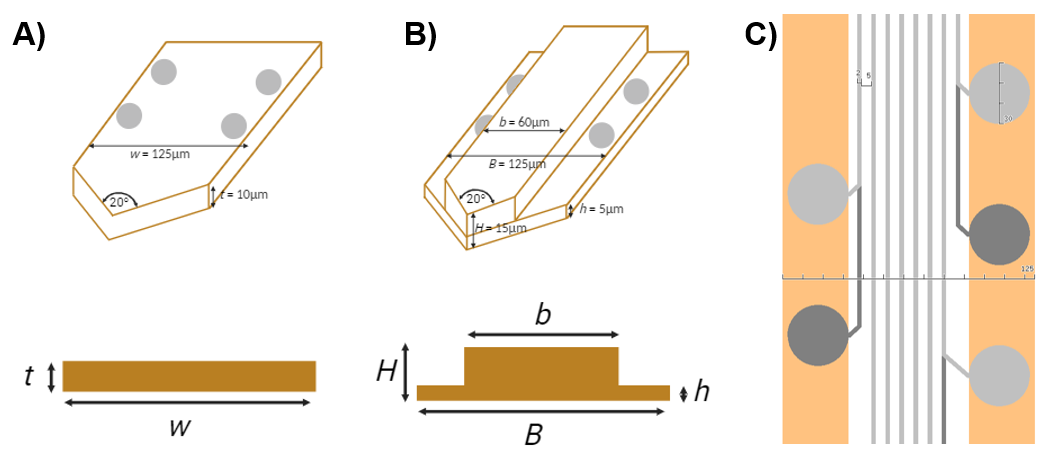


Figure S2: Illustration of the design parameters. A) conventional flat MEA and B) BRATS MEA. C) Trace width and spacing as well as the electrode size and shank width.


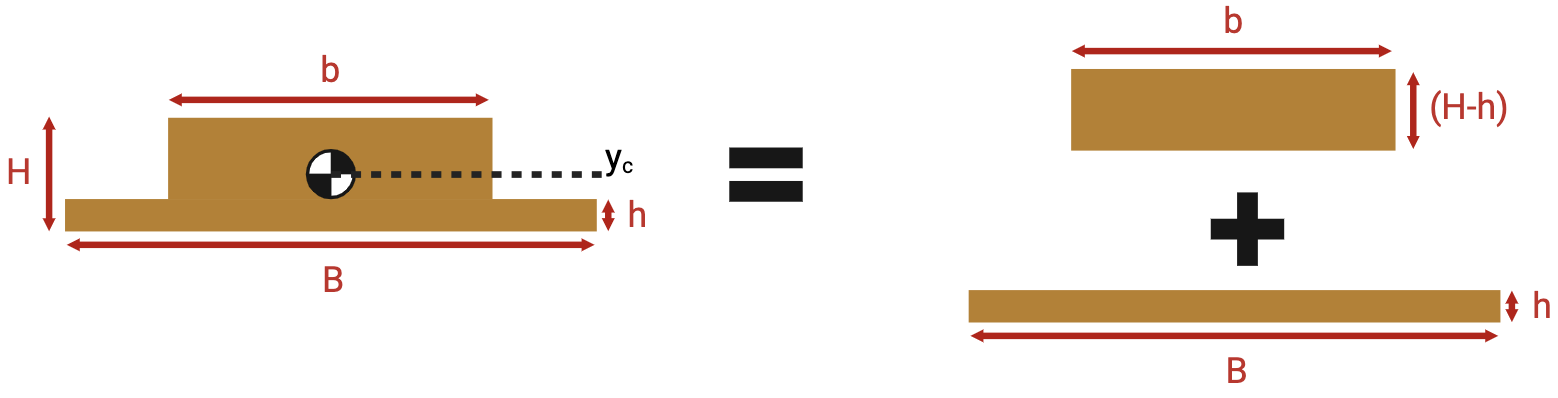


I_1_

I_2_

**Figure S3: Moment of inertia of BRATS MEA.**

Centroid of BRATS MEA: $y_{c}= \frac{\sum\overline{y}A}{\sum A}=\frac{\frac{h}{2}.Bh+\frac{H-h}{2}.b(H-h)}{Bh+b(H-h)}$

Consider the moment of inertia I of BRATS MEA as the sum of the moments of inertia of two parts ($I_{1} + I_{2}$) about $y_{c}$. Using the parallel axis theorem:

$$I_{1}= \frac{b\left( H-h \right)^{3}}{12}+b\left( H-h \right)\left( y_{c}-\frac{H+h}{2} \right)^{2}$$

$$I_{2}=\frac{Bh^{3}}{12}+Bh\left( y_{c}-\frac{h}{2} \right)^{2}$$

Therefore,

$$I=I_{1}+I_{2}=\frac{Bh^{3}}{12}+Bh\left( y_{c}-\frac{h}{2} \right)^{2}+\frac{b\left( H-h \right)^{3}}{12}+b\left( H-h \right)\left( y_{c}-\frac{H+h}{2} \right)^{2}$$

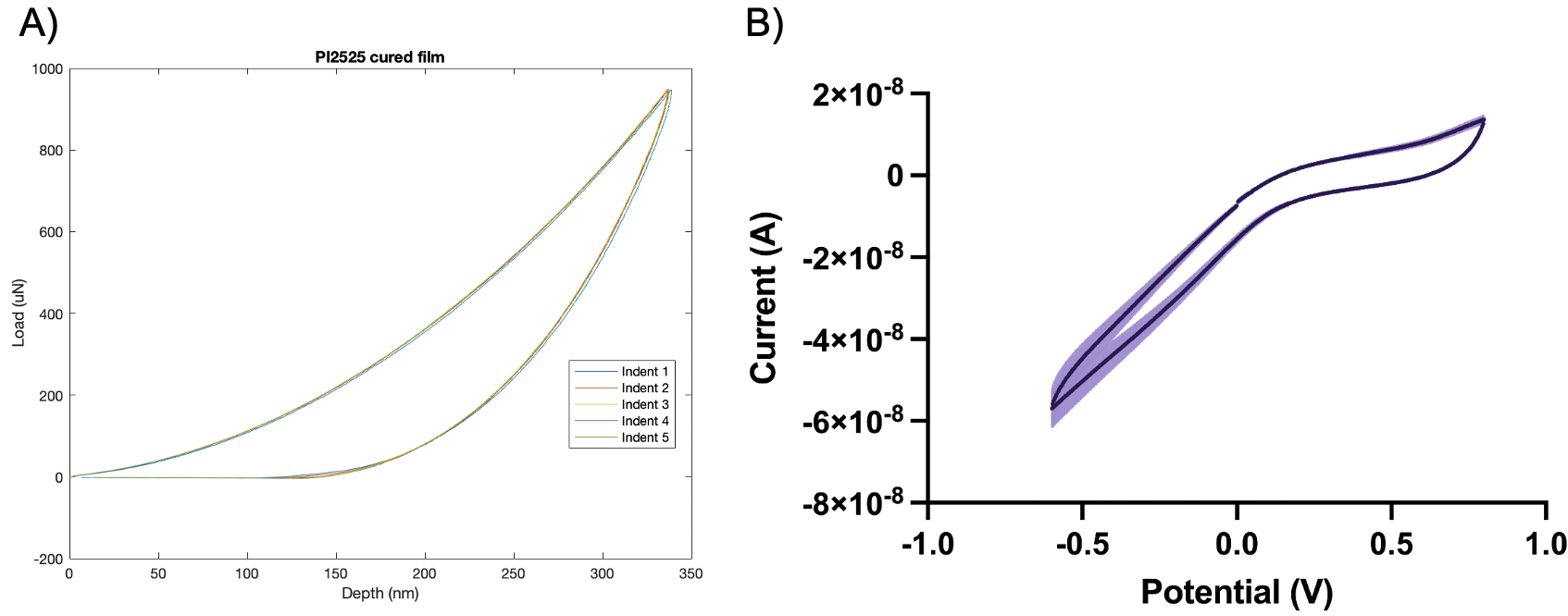


Figure S4: Additional characterizations. A) Load vs depth curve from 5 nanoindents on cured PI2525 film. B) Cyclic voltammogram of a representative BRATS MEA with Pt sites (mean ± SEM).


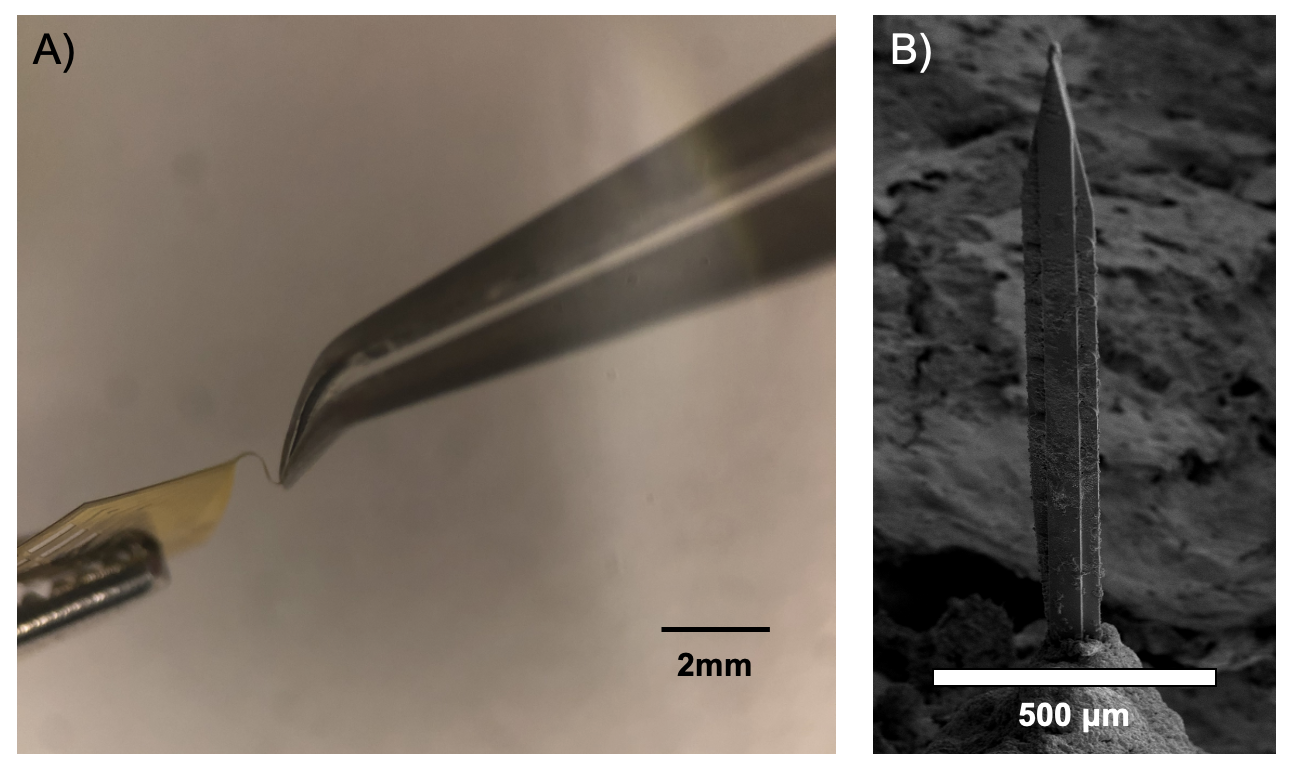


Figure S5: Flexibility and durability of BRATS MEAs. A) Demonstration of the flexibility of the BRATS MEA shank. B) Scanning electron microscopy showing intact BRATS MEA explanted after 1 week of implantation in rat brain.


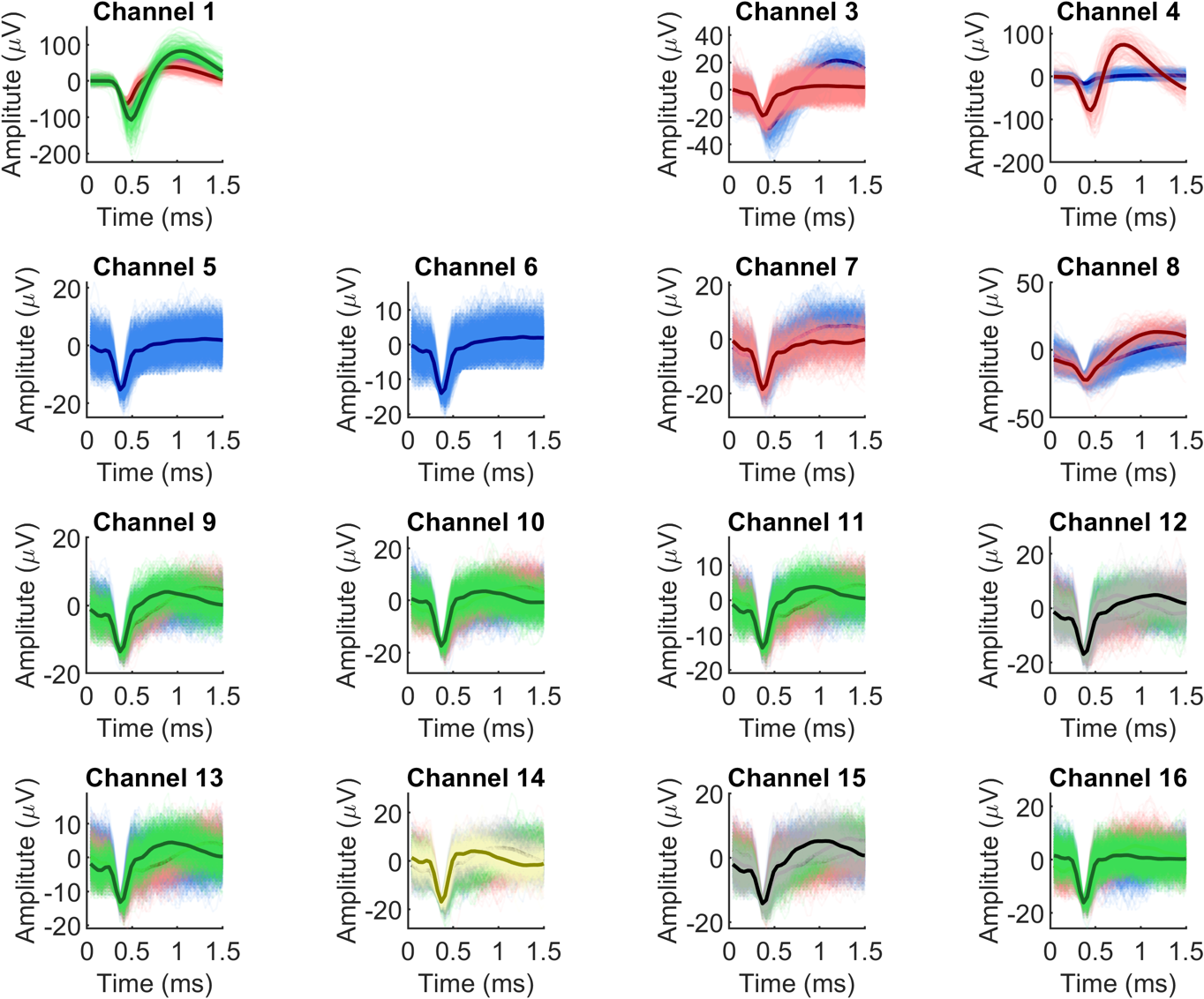


Figure S6: Single and multi-unit activities recorded from the BRATS MEA during an acute recording session from rat cortex.


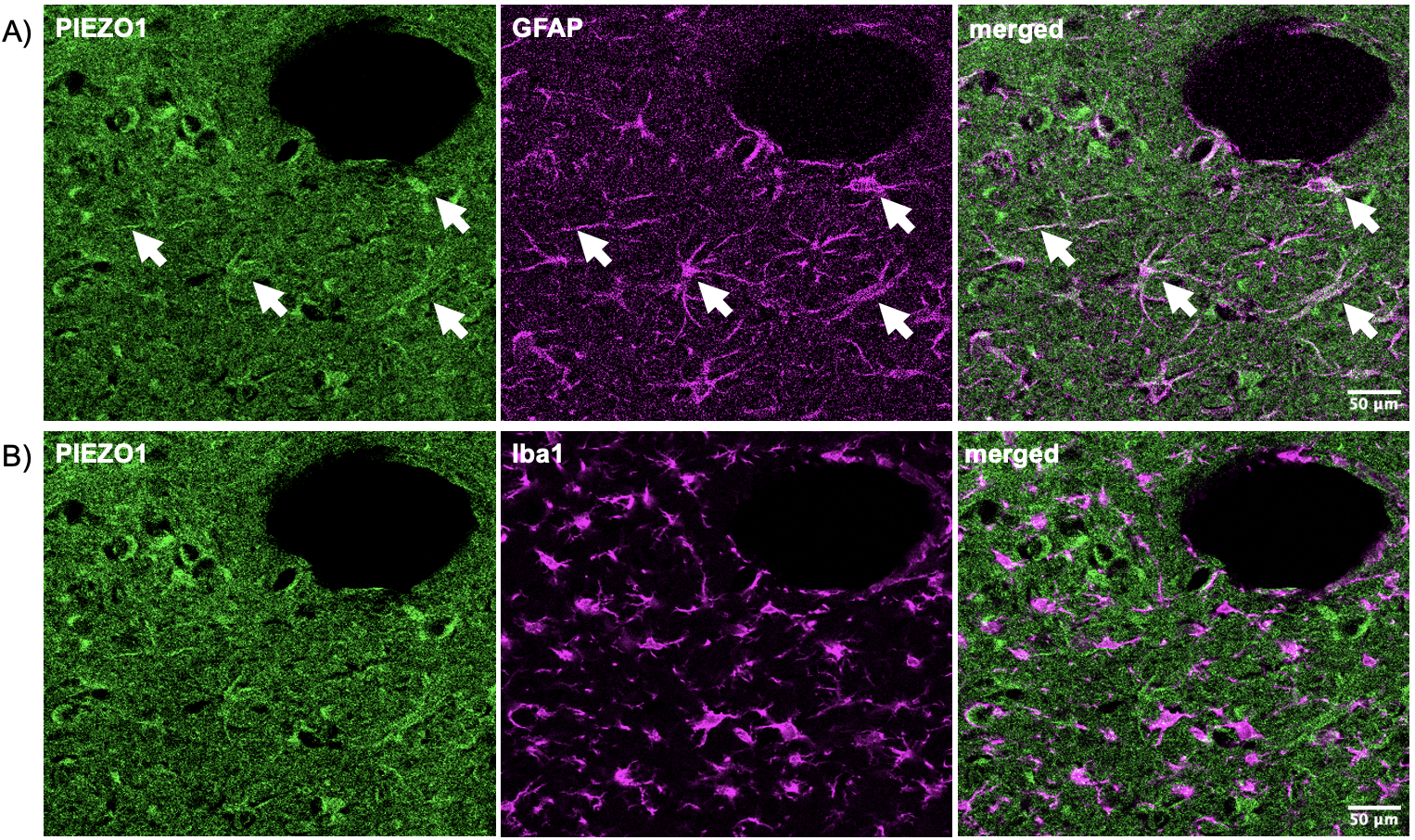


Figure S7: PIEZO1 colocalization in glia cells. A) PIEZO1 and GFAP colocalization showing astrocytic PIEZO1 (white arrows). B) Little colocalization of PIEZO1 observed in Iba-1
